# Effectiveness of a Videoconference-Based Cognitive Behavioral Therapy Program for Patients with Schizophrenia: Pilot Randomized Controlled Trial

**DOI:** 10.2196/59540

**Published:** 2025-01-14

**Authors:** Masayuki Katsushima, Hideki Nakamura, Yuki Shiko, Hideki Hanaoka, Eiji Shimizu

**Affiliations:** 1 Department of Rehabilitation, Faculty of Health Care and Medical Sports Teikyo Heisei University Ichihara Japan; 2 Research Center for Child Mental Development Chiba University Chiba Japan; 3 Department of Nursing, Faculty of Medicine The Jikei University School of Medicine Tokyo Japan; 4 Department of Cognitive Behavioral Physiology Chiba University Graduate School of Medicine Chiba Japan; 5 Department of Biostatistics, Graduate School of Medicine Saitama Medical University Saitama Japan; 6 Clinical Research Center Chiba University Hospital Chiba Japan; 7 Cognitive Behavioral Therapy Center Chiba University Hospital Chiba Japan

**Keywords:** schizophrenia, randomized controlled trial, cognitive behavioral therapy, videoconference, remote therapy

## Abstract

**Background:**

Cognitive behavioral therapy for psychosis (CBTp) is not sufficiently widespread in clinical practice, although evidence has been presented.

**Objective:**

The purpose of this study was to explore whether one-on-one videoconference-based CBTp (vCBTp) is more effective than usual care (UC) alone for improving psychiatric symptoms in patients with schizophrenia attending outpatient clinics.

**Methods:**

In this exploratory randomized controlled trial, patients with schizophrenia and schizoaffective disorders who were still taking medication in an outpatient clinic were randomly assigned to either the vCBTp plus UC group (n=12) or the UC group (n=12). The vCBTp program was conducted once a week, with each session lasting for 50 minutes, for a total of 7 sessions conducted in real-time and in a one-on-one format remotely using a loaned tablet computer (iPad). The primary outcome was the Positive and Negative Syndrome Scale (PANSS) total score, which measures the difference in the mean change from baseline (week 0) to posttest (week 8).

**Results:**

The study included 24 participants. There were no significant differences between the 2 groups at baseline. With regard to significant differences between the 2 groups in terms of the primary outcome, the mean change in the PANSS total score from baseline to week 8 in the vCBTp plus UC group was –9.5 (95% CI –12.09 to –6.91) and the mean change in the UC alone group was 6.9 (95% CI 1.54-12.30). The difference between the 2 groups was significant (*P*<.001). In addition, significant improvements were observed in the subscales of positive (*P*<.001) and negative (*P*=.004) symptoms and general psychopathology (*P*<.001). Significant differences were also observed in the secondary outcomes of the General Anxiety Disorder-7 (GAD-7; *P*=.04) and EQ-5D-5L (*P*=.005). There were no dropouts and no serious adverse events in this study.

**Conclusions:**

A total of 7 remote vCBTp sessions conducted in the vCBTp plus UC group could be safely administered to patients with schizophrenia. They were also observed to be effective for psychiatric symptoms, general anxiety, and quality of life. However, because of the observed worsening of scores in the UC group, caution is required in interpreting significant differences between the 2 groups. This approach is expected to improve accessibility to CBTp for outpatients with schizophrenia and social anxiety regarding transportation use and financial and physical burdens related to transportation, and to contribute to promoting CBTp acceptability by compensating for the shortage of implementers.

**Trial Registration:**

University Hospital Medical Information Network Clinical Trials Registry UMIN000043396; https://center6.umin.ac.jp/cgi-open-bin/ctr/ctr_view.cgi?recptno=R000049544

**International Registered Report Identifier (IRRID):**

RR2-10.1136/bmjopen-2022-069734

## Introduction

### Background

Schizophrenia is a psychiatric disorder that affects approximately 1% of the total population and is typically prevalent between the ages of 16 and 30 years [1,2]. Pharmacotherapy with antipsychotic drugs is the standard treatment for schizophrenia. Moreover, cognitive behavioral therapy, a psychological intervention, is recommended by the National Institute of Health and Clinical Excellence (NICE) [3]. In the West, cognitive behavioral therapy for psychosis (CBTp) has shown promise in improving positive symptoms and acquiring coping skills [4]. A meta-analysis of CBTp reported a moderate effect of 0.35-0.44 for positive and generalized symptoms [5,6]. Despite the effectiveness of traditional face-to-face CBTp in treating schizophrenia, progress in training therapists to conduct CBTp has been slow [7,8]. The Schizophrenia Commission in the United Kingdom reported that only 10% of patients with schizophrenia receive CBTp due to limited access [9]. Therefore, despite supporting evidence, it is infrequently implemented in clinical practice owing to a shortage of practitioners, making it difficult for patients to receive CBTp [10]. It is thus desirable to create an environment in which effective CBTp can be implemented in the future and increase the number of clinicians who can practice. Evidence of low-intensity CBTp has also been collected, suggesting its efficacy [11,12].

Previous studies have found that community psychiatric nurses effectively increased their awareness of illnesses by conducting as few as 6 sessions of CBTp [13,14]. The 6 CBTp sessions improved their understanding of illness, general health, and depression. NICE recommends a minimum of 16 sessions of CBTp [3]. However, a meta-analysis by Hazell et al [11] reported an effect size of 0.46 for CBTp conducted in 6-15 sessions.

In addition, some studies have reported the incorporation of new theories, such as mindfulness, acceptance and commitment therapy, compassion mind training, and metacognitive therapy, into CBTp [15]. Thus, in recent years, studies have been conducted that have not limited themselves to formulated CBTp but have adjusted the frequency and content of CBTp.

In this context, studies reported that patients with schizophrenia have traumatic memories and may be treated with CBT [16]. However, CBTp does not focus directly on the sequelae of trauma, and therapists are concerned that experiential reprocessing of trauma may exacerbate psychotic symptoms; therefore, the treatment of posttraumatic disorder (PTSD) symptoms often involves reluctance [17]. Nevertheless, 3 reviews [18-20] suggested that trauma-focused psychotherapy is safe and effective for psychotic patients.

In recent years, research on internet-based CBTp has increased [21-25], and a growing interest exists for CBT delivered through a videoconference system called vCBT [26,27]. This method enhances access to CBT for patients in remote areas and enables teletherapy through 2-way real-time communication between the therapist and patient. Therapist-led internet-based CBT has been demonstrated to be equally effective as face-to-face CBT in treating anxiety, depression, insomnia, and physical disorders [28].

There have also been several previous studies targeting schizophrenia. A CBTp effort in a group therapy format using a videoconference method for early psychosis reported positive results regarding efficacy [29]. Moreover, a study of remote sessions based on acceptance commitment therapy in a group therapy format mentioned the potential for both accessibility and recovery effects [30]. In addition, there have been reports of neurocognitive and metacognitive training delivered remotely as group therapy [31]. However, there is still little to no evidence regarding randomized controlled trials (RCTs) of CBTp interventions in a one-on-one videoconference format.

Therefore, we developed a real-time and one-on-one videoconference-based CBTp (vCBTp) program for schizophrenia through an RCT design [32]. The vCBTp program included 2 sessions that addressed intrusive memories of stressful events related to the onset of psychosis.

### Objective

This study examined the results and efficacy of an exploratory RCT designed to compare the clinical efficacy of 7 sessions of vCBTp as an adjunct to usual care (UC) versus UC alone. The study included patients with persistent positive symptoms of schizophrenia despite pharmacotherapy.

## Methods

### Study Design and Participants

This study was designed as a single-center, 2-arm, parallel, rater-blinded, prospective RCT with a 7-week treatment regimen. Study participants were assigned to either the vCBTp plus UC or UC alone groups. The recruitment of participants was posted on the Chiba University Graduate School of Medicine’s Cognitive Behavioral Physiology subject recruitment website. Recruitment posters were displayed in the outpatient waiting room of Chiba University Hospital. Recruitment posters and leaflets were also mailed to medical institutions and employment support facilities in Chiba Prefecture.

### Ethical Considerations

All procedures were approved by the Institutional Review Board of Chiba University Hospital in January 2021 (reference number: G2020031). All participants received a thorough verbal and written explanation of the study, and verbal and written consent was obtained from those who were confirmed to have the capacity to consent to the study and who wished to participate. Participation in the clinical trial was voluntary on the part of the subjects and their surrogates, and they were fully informed that they could refuse or withdraw from participation in the clinical trial at any time. The consent obtained also covered that no additional consent would be required for secondary analyses.

Data were anonymized so that they could be identified only by a unique identification number and were maintained at the data center. Consent documents and other materials related to the study were strictly maintained in a locked archive.

Study participants were paid a burden-reduction fee (10,000 JPY [US $65] gift card at baseline and 10,000 JPY [US $65] gift card at the endpoint for a total of 20,000 JPY [US $130]) for assessments at 0 and 8 weeks. This burden-reduction fee was paid equitably to both the vCBTp plus UC group and the UC group.

### Inclusion Criteria

The criteria for those eligible to participate in the study were as follows: age 16-65 years; primary diagnosis of schizophrenia, schizoaffective disorder, or delusional disorder according to the Diagnostic and Statistical Manual of Mental Disorders, 5th edition; and satisfactory assessment of competence to provide informed consent using the MacArthur Competence Assessment Tool for Clinical Research (MacCAT-CR) [33,34]. The MacCAT-CR assessed participants’ ability to “understand,” “recognize,” “think validly,” and “express choice” about the study. Participants who responded with a score of 0 on any of the items were judged to have poor capacity to consent and were eligible for exclusion. In addition, participants needed to have at least 3 points on any one of the positive symptoms of the Positive and Negative Syndrome Scale (PANSS) [35]. In addition, participants had to be attending their primary psychiatrist, be taking antipsychotic medication for at least 3 months continuously, and not intend to change their medication within the next 3 months. Participants were also required to obtain consent from their primary psychiatrist to participate in the study. An internet environment with wireless fidelity (Wi-Fi) was needed for videoconference using a tablet computer (iPad).

### Exclusion Criteria

Those with a diagnosis of alcoholism, drug dependence, intellectual disability, or dementia from their primary psychiatrist were excluded from participation in the study. Those who were predicted to be at risk of self-harm or harm as a result of the clinical trial were also excluded. Risk of self-harm or harm was assessed by responses to outcome items and observation of adverse events at the time of consent and at 0- and 8-week assessments. In addition, patients were screened for suicidal ideation with item 9 of the Patient Health Questionnaire-9 (PHQ-9) [36] at the 0- and 8-week assessments; those scoring 1 or more points were considered positive for suicidal ideation [37]. Patients were assessed by the investigator and a psychiatrist, and those who were assessed as being at imminent risk of suicide and thus unable to complete the study were considered for exclusion. Patients who were hospitalized were excluded from the study.

### Sample Size

The main objective of this study was to assess the superiority of vCBTp for symptom improvement in a group of patients with schizophrenia compared with a UC group, referencing a previous study by Morrison et al [38], which compared CBT and antipsychotic groups, where the mean difference between the vCBTp plus UC group and UC group after the intervention was 12 points, with the SD of the pooled UC group assumed to be 8 points. The significance level for both groups was set at 5%, and the power was set at 90%. The number of patients in each group was calculated to be 11. Assuming a participant dropout rate of 10%, the target number of patients was set at 12 in each group.

### Eligibility Assessment

Two researchers (a psychiatrist [ES] and a researcher [MK]) conducted the initial consultation. It was conducted face-to-face at Chiba University Hospital. At the hospital, ES, as a psychiatrist, assessed and confirmed eligibility, including the participant’s history of treatment by their family physician, severity of symptoms, and ability to consent to participate in the study. MK assessed the PANSS for allocation adjustment factors and the MacCAT-CR for competency to consent to this study. In addition, the researchers ascertained the participants’ clinical characteristics, including gender, age, marital status, age at onset, duration of illness, employment status, and treatment history (eg, names of antipsychotic medications, drug titers at baseline, and changes in treatment during the study period).

### Randomization and Masking

After ES or MK examined applicants for suitability prior to study enrollment, those who were identified as meeting the criteria and whose consent was obtained were enrolled as cases by the Chiba University Hospital Data Center. After registration, all patient names were key-coded, anonymized, and kept confidential. The Chiba University Hospital Data Center then conducted dynamic allocation of participants using a computer program. Because this was a pilot study and the number of patients was small, 2 allocation adjustment factors were used. Participants were randomized in a 1:1 ratio to the vCBTp plus UC group or UC group using a minimization method with 2 factors as allocation adjustment factors: total baseline PANSS score (PANSS ≥ 51) and gender. The PANSS values were calculated based on the participants’ baseline values in the study by Naeem et al [39]. Each participant was randomly assigned to 1 of the 2 treatments. In addition, the data center was strictly controlled to ensure that the allocation program was not divulged to outside parties. After study entry, the author HN, as an evaluator, was blinded for the duration of the study, and only HN who was an expert in assessing the PANSS at 0 and 8 weeks assessed the PANSS in a semistructured interview format via videoconference. HN was not informed which group participants were assigned to until study completion to ensure fairness. All secondary assessments other than the PANSS involved self-administered questionnaires.

### Procedures

Patients who wished to participate in the study had an initial consultation with MK and ES at Chiba University Hospital, where eligibility assessment and consent to participate in the study were explained. At that time, they received an explanation and lecture on the basic operation of the loaned iPad and videoconference connection for vCBTp. With regard to vCBTp, MK participated from Chiba University Hospital, and patients attended sessions from home using the loaned iPad. Participants reported their email address and cell phone number to the study office for contact, but were also required to provide emergency contact information for a family member or support person in case of emergency. Videoconference was used for 0- and 8-week assessments and all vCBTp sessions. The PANSS assessments at weeks 0 and 8 were conducted via videoconference with only HN and the participants. MK did not participate in the videoconference.

All vCBTp sessions were conducted by MK. ES appropriately supervised MK after each session. In addition to research experience in CBT interventions for schizophrenia [40], MK had conducted high-intensity CBT sessions for depression and anxiety at the Cognitive Behavioral Therapy Center at Chiba University Hospital, and had the qualities and fidelity of a therapist.

ES had experience in creating remote CBT programs for patients with panic disorder, obsessive-compulsive disorder, social anxiety [26], eating disorders [41], chronic pain [42], and other disorders. The vCBTp program for patients with schizophrenia was created by 2 researchers (ES and MK). The number of sessions in this study was set based on a meta-analysis of low-intensity CBTp [11]. Since the median number of sessions in the 10 previous studies in the meta-analysis was 7.5, the program was created with a total of 7 sessions of vCBTp. The duration of treatment was 7 weeks (50 minutes per session, once a week). Seven sessions were printed as text in a paper booklet and mailed to the vCBTp plus UC group participants after the 0-week assessment. vCBTp sessions were conducted in a one-on-one format between the therapist and the participant. The therapist wrote the session content on slides in real-time while the camera was turned on and a PowerPoint file was shared on the screen in the messaging application software (Microsoft Teams) for videoconference. After each homework session, participants wrote on the text file that had been sent to them in advance and submitted the images to the therapist as email attachments with photos.

In schizophrenia, depression and anxiety are significantly related to positive symptoms [43]. We developed the vCBTp sessions based on “cognitive restructuring,” a basic technique incorporated into CBT for depression and anxiety. vCBTp consists of the following 7 sessions: (1) assessment and goal setting, (2) case formulation using cognitive-behavioral models, (3) cognitive restructuring, (4) coping and relaxation, (5) externalization of intrusive memories about stressful events related to the onset of psychosis, (6) re-evaluation of intrusive memories about stressful events related to the onset of psychosis, and (7) relapse prevention (Table 1).

**Table 1:** Composition and contents of videoconference-based cognitive behavioral therapy for psychosis.

Session	Contents and goals	Homework
Session 1	Assessment and goal setting; identify symptoms of distress and set goals	Record stressful situations felt during the week
Session 2	Case formulation of current stress; use the cognitive-behavioral model to objectively understand events and thoughts/anxiety	Cognitive-behavioral model to record stressful situations felt during the week
Session 3	Cognitive restructuring of current stress; psychological flexibility by mentioning “other ideas;” improvement of confidence	Use the column method to derive and record another way of thinking
Session 4	Coping and relaxation; engage in fun and calming activities and activate behaviors	Record coping done in a week
Session 5	Externalize intrusive memories about stressful events related to the onset of psychosis; use cognitive-behavioral models to capture past stressful experiences and understand core beliefs that have persisted since that time; moreover, engage in self-compassion at that time	Record words of self-compassion about your symptoms and stress felt during the week
Session 6	Reassess intrusive memories about stressful events related to the onset of psychosis; reflect on how you have coped with past stressful experiences and recognize that you have coping skills	Record your coping behaviors about symptoms and stress felt during the week
Session 7	Prevention of recurrence, review of the past session theme, and setting of future goals	Fill in what you learned or felt during the vCBTp^a^ session

^a^vCBTp: videoconference-based cognitive behavioral therapy for psychosis.

We also focused on intrusive thoughts and subjective distress caused by broadly defined traumatic stress experiences in patients with schizophrenia [44-46]. The Graduate School of Medicine, Chiba University, to which the researcher belongs, also has a track record of research on imagery rescripting of traumatic memories [47]. However, in this study, instead of dealing with “traumatic memories” in consideration of the burden on participants, the aim was to help participants become aware of their core beliefs and work on themselves by reflecting on stressful events related to their past onset of psychosis. The fact that the research sessions would include an important session in which participants would reflect on painful events in their past was fully explained and agreed upon during the study description. It was also decided that sessions would focus on episodes that participants felt comfortable talking about. The order of sessions was flexible, with sessions being changed or repeated depending on the participants’ levels of understanding and their reactions at the time.

There was no staff involvement in this study with regard to UC, which was defined as participants’ own use of outpatient care and rehabilitation that they were engaged in prior to participation in the study and until its conclusion.

While use anxiety and connection problems are possible with the use of digital devices, the study addressed these by providing the following support: vCBTp patients practiced connecting to MK for participation through videoconference from home prior to the 0-week baseline assessment. vCBTp patients were also provided with an iPad charger and a microphone with an earpiece for consideration of their family living together. Patients with no or unstable home Wi-Fi access were loaned mobile Wi-Fi at no charge. While participating in the study, they could be contacted at any time via email. Participants were able to contact the researcher via email if they caught a cold or had other urgent appointments. When connected, participants were also called as needed to provide support for iPad use and remote connection anxiety, as appropriate. In addition, after each session was completed for the vCBTp plus UC group, the materials from that session were sent in PDF format via email. After the research trial was completed, the PowerPoint presentation materials for the participants, which had been included during 7 sessions, were printed as a paper-based booklet for the participants’ own use and mailed to them by the researcher.

### Outcomes

#### Primary Outcome

The primary outcome was the PANSS total score. The change from baseline to week 8 was compared. The PANSS is an assessment consisting of 7 positive symptoms, 7 negative symptoms, and 16 general psychopathology items. Each item is scored on a scale of 1-7 points. The scores range from 30 (no symptoms) to 210 (the most severe symptoms). The PANSS is an objective instrument for assessing schizophrenia symptoms in clinical and experimental studies and is the global standard for reliability and validity. Assessors blinded to the treatment assignment assessed the PANSS via a videoconference system at baseline and 8 weeks after the test.

#### Secondary Outcomes

Subtotal scores were assessed for positive symptoms, negative symptoms, and general psychopathology extracted from the PANSS subscale scores. Total scores for the 7 positive symptom subscales of the PANSS ranged from 7 (no symptoms) to 49 (severe positive symptoms). Total scores for the 7 negative symptom subscales ranged from 7 (no symptoms) to 49 (severe negative symptoms). Total scores for the 16 items in the general psychopathology subscale ranged from 16 (no symptoms) to 112 (severe psychopathology).

The Japanese version of the Beck Cognitive Insight Scale (BCIS-J) [48] was also used to assess cognitive insight. All 15 items of the BCIS-J were divided into 9 items of self-reflection (0-36) and 6 items of confidence (0-24). A lower subtotal of the 9 self-reflection items minus the 6 confidence items is associated with a lower cognitive pathology score.

In addition, the Impact of Event Scale-Revised (IES-R) [49] was used to assess subjective distress over specific stressful life events. The IES-R consists of 22 items (0-88) that are divided into 8 intrusive symptoms, 8 avoidance symptoms, and 6 hyperarousal symptoms, measuring the symptoms of persons exposed to traumatic experiences.

The PHQ-9 was used to assess depressive symptoms. It consists of 9 items ranging from 0 (no depressive symptoms) to 27 (severe depressive symptoms).

The Generalized Anxiety Disorder-7 (GAD-7) [50] was used to assess anxiety. The GAD-7 consists of 7 items ranging from 0 (no anxiety symptoms) to 21 (severe anxiety symptoms).

The EQ-5D-5L [51] was used to assess quality of life (QoL). It consists of 5 items and assesses QoL on a 5-point Likert scale ranging from 1 (not severe) to 5 (severe). The EQ-5D-5L is the most commonly used measure of QoL worldwide.

#### Other Outcomes

Other assessments included chlorpromazine (CPZ) [52], which indicates (equivalent to) the daily prescribed dose of antipsychotic medication. In addition, the therapists inquired about participants’ experiences of adverse events during each assessment. All measures were assessed at week 0 (baseline) and week 8 (after treatment), and the results were analyzed according to set principles.

### Statistical Analyses

This study followed the Consolidated Standards of Reporting Trials (CONSORT) guidelines for statistical analyses and reporting. The primary analysis was based on the intention-to-treat principle. We calculated the PANSS total score change at week 8 for both the vCBTp and UC groups and compared the mean differences between the groups to assess the primary outcome. Comparisons between the 2 groups were performed using an unpaired *t* test. Secondary endpoints were analyzed using the same approach as the primary endpoint to understand the primary endpoint further. In addition, we considered participants in each group with a 20% improvement in the total PANSS score as treatment responders. We compared the proportion of participants in each group. The frequency of adverse events was used as a safety endpoint. For percentage estimates, the exact 2-sided 95% CIs of the binomial distribution were calculated for each group. Statistical analyses were performed using SAS version 9.4 (SAS Institute Inc). Statistical significance was set at a *P*-value of <.05. The Cohen *d* (M baseline–M posttest)/SD pooled formula was used to calculate the effect size (Cohen *d*) from the difference in the means of each group.

## Results

### Recruitment

**Figure 1:**
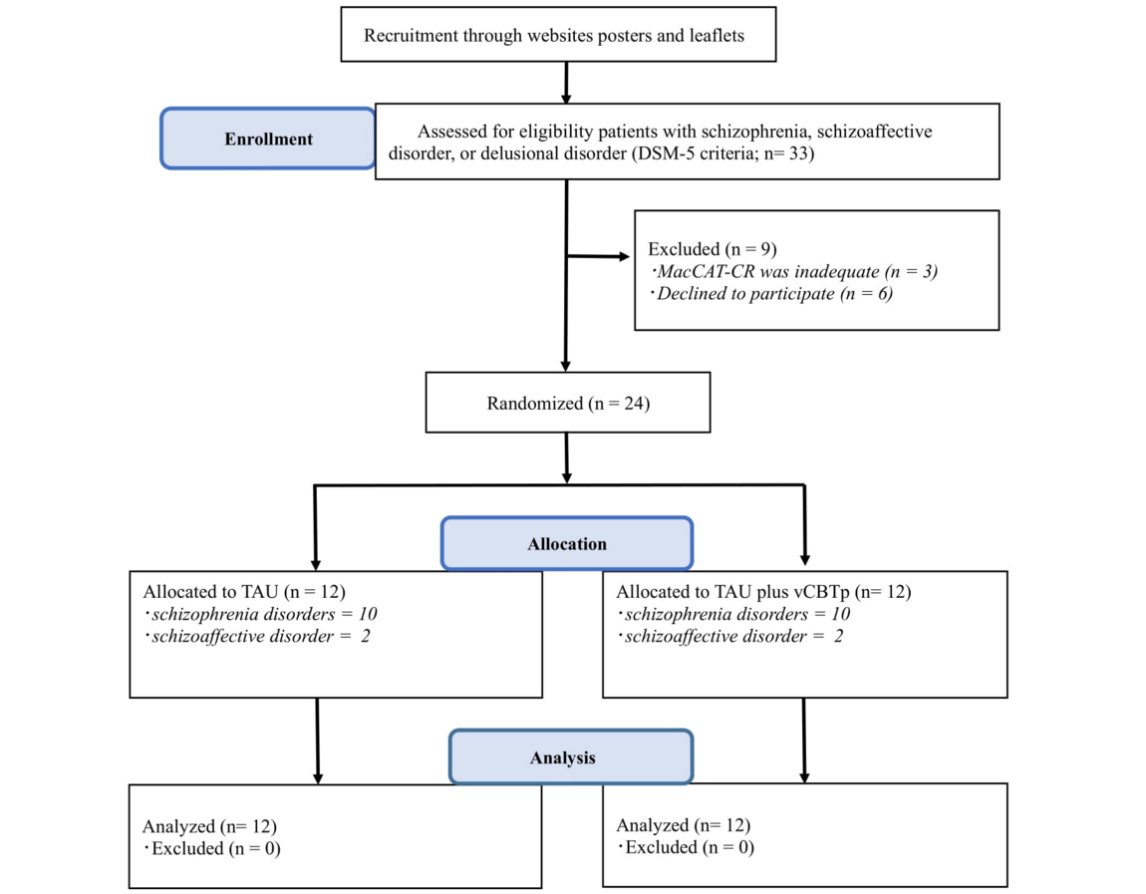
Flow diagram of patient recruitment. DSM-5: Diagnostic and Statistical Manual of Mental Disorders, 5th edition; MacCAT-CR: MacArthur Competence Assessment Tool for Clinical Research; UC: usual care; vCBTp: videoconference-based cognitive behavioral therapy for psychosis.

Figure 1 shows the flow diagram of patient recruitment based on the CONSORT guidelines. A total of 33 patients applied to participate. Applicants included those who applied through the website (n=5), those who saw the poster in the hospital outpatient waiting room and applied on their own (n=4), and those who were referred by their doctor or daycare facility staff (n=14). Three of these patients were excluded. Of the 3 excluded patients, 1 was excluded because of hospitalization. The other 2 patients were excluded because they had multiple zeros on the MacCAT-CR, did not meet the criteria for understanding the trial, and were found not to have adequate capacity to consent. These 2 individuals were referred by a daycare facility staff member, who did not have a good understanding of the clinical trial and was primarily interested in obtaining the burden-reduction funding. In addition, 6 patients voluntarily declined to participate in the study after being informed about the study, citing concerns about the burden of the study and fatigue. Ultimately, 24 patients underwent in-person eligibility examinations at Chiba University Hospital, and all 24 were enrolled in the study. Study participants had schizophrenia (n=18) and schizoaffective disorder (n=4) and were assigned in equal numbers to the 2 groups. All 24 participants in the assigned vCBTp plus UC and UC groups completed the clinical trial. They provided informal feedback on the vCBTp plus UC intervention. Nearly all the study participants who completed the study reported positive feedback, indicating that the intervention was appropriate and easy to engage in (Figure 1).

### Participants’ Characteristics

Table 2 summarizes the clinical characteristics of the participants at the time of assignment. There were no significant differences between the 2 groups in terms of age, gender, marital status, employment status, number of people living together, number of psychiatric hospitalizations, age of onset, duration of illness, total MacArthur comprehension score, and PANSS participant proportion score below 50 (Table 2).

**Table 2:** Baseline characteristics of the study participants (N=24).

Variable	vCBTp^a^ + UC^b^ group (n=12)	UC group (n=12)
Female, n (%)	7 (58)	7 (58)
Unmarried, n (%)	8 (89)	9 (75)
Unemployed, n (%)	10 (83)	9 (75)
Age (years), mean (SD)	34.7 (8.98)	32.3 (12.26)
Hospitalization (times), mean (SD)	1.6 (1.44)	1.3 (1.87)
Age of onset (years), mean (SD)	22.8 (6.70)	24.4 (8.26)
Duration of an illness (years), mean (SD)	11.6 (7.44)	7.6 (5.74)
MacCAT-CR^c^ (total score), mean (SD)	41.1 (1.11)	40.3 (1.84)
Number of patients with a PANSS^d^ score less than 51, n (%)	3 (25)	2 (17)

^a^vCBTp: videoconference-based cognitive behavioral therapy for psychosis.

^b^UC: usual care.

^c^MacCAT-CR: MacArthur Competence Assessment Tool for Clinical Research.

^d^PANSS: Positive and Negative Syndrome Scale.

Participants were mostly single (n=17) and unemployed (n=19). They were in their early 30s on average, and the average number of hospitalizations was less than two, although some had never been hospitalized (n=5). Some respondents were living alone in apartments (n=1) or in assisted living (n=1), but the rest were living with family members (n=22). An equal number of patients with schizophrenia (n=10) and schizoaffective disorder (n=2) participated in each group. These ambulatory variables were not used as randomization factors.

In addition, all participants had a certain affinity for digital devices, as they usually used smartphones and PCs to watch videos. Both groups were able to maintain a remote connection environment without major problems by exchanging emails and receiving advice over the phone in case of connection problems.

### Primary Outcome

Table 3 displays the means and SDs of the participants’ PANSS total scores from baseline to posttest. The mean change in PANSS total scores from baseline to posttest was –9.5 (95% CI –12.09 to –6.91) for the vCBTp plus UC group and 6.9 (95% CI 1.54-12.30) for the UC group (Table 4). The vCBTp plus UC group demonstrated a significant improvement compared to the UC group, with a between-group difference of 16.41 (95% CI 10.79-22.04; *P*<.001).

**Table 3:** Raw data of the primary and secondary outcomes (N=24).

Variable					vCBTp^a^ + UC^b^ group (n=12)										UC group (n=12)					
					Pre score, mean (SD)			Post score, mean (SD)			Cohen *d*			Pre score, mean (SD)				Post score, mean (SD)		Cohen *d*
**Primary outcome**																				
	PANSS^c^ (total score)			52.3 (7.02)			42.8 (6.02)			1.45			54.2 (11.07)				61.1 (10.72)			–0.61
**Secondary outcomes**																				
	**PANSS (positive)**			14.8 (2.77)			11.4 (1.88)			1.43			13.7 (3.77)				14.6 (3.50)			–0.24
		Delusion	3.4 (1.31)			2.7 (0.78)			0.65			3.5 (1.45)				3.6 (1.31)			–0.07	
		Conceptual disorganization	1.8 (0.75)			1.4 (0.51)			0.62			1.6 (0.90)				1.8 (0.83)			–0.23	
		Hallucinatory behavior	1.8 (1.11)			1.6 (0.67)			0.22			2.3 (1.30)				2.8 (1.42)			–0.37	
		Excitement	1.8 (1.29)			1.3 (0.45)			0.52			1.2 (0.39)				1.2 (0.39)			0.00	
		Grandiosity	1.8 (1.54)			1.3 (0.45)			0.35			1.5 (1.00)				1.4 (0.79)			0.11	
		Suspiciousness	3.2 (1.53)			2.3 (0.97)			0.70			2.6 (1.16)				2.8 (0.87)			–0.20	
		Hostility	1.1 (0.29)			1.0 (0.00)			0.14			1.0 (0.00)				1.1 (0.29)			–0.13	
	PANSS (negative)			10.8 (2.63)			9.6 (1.78)			0.53			13.7 (6.87)				16.3 (5.03)			–0.43
	**PANSS (general psychopathology)**			26.8 (4.18)			21.8 (2.96)			1.38			26.8 (4.57)				30.2 (5.65)			–0.66
		Anxiety	2.8 (1.11)			2.0 (0.60)			0.90			2.8 (1.19)				2.8 (1.19)			0.00	
		Guilt feeling	2.9 (1.38)			2.1 (0.90)			0.69			2.1 (0.67)				2.3 (0.75)			–0.28	
		Depression	2.1 (1.00)			1.6 (0.67)			0.59			2.0 (1.04)				2.8 (1.40)			–0.42	
		Lack of judgment and insight	1.9 (0.90)			1.4 (0.51)			0.68			2.1 (0.79)				2.3 (0.87)			–0.24	
	BCIS-J^d^ (self-certainty)			4.7 (2.27)			4.2 (2.41)			0.21			7.0 (3.28)				7.9 (5.05)			–0.21
	BCIS-J (self-reflectiveness)			14.5 (3.12)			16.7 (3.85)			0.63			11.6 (1.19)				12.8 (4.67)			0.35
	**IES-R^e^ (total score)**			37.1 (13.43)			31.8 (14.68)			0.36			30.3 (16.55)				32.1 (16.08)			–0.11
		Intrusion	13.3 (5.38)			10.0 (4.88)			0.64			11.8 (9.36)				11.3 (7.81)			0.06	
		Avoidance	15.3 (4.59)			14.2 (6.28)			0.20			12.2 (7.90)				12.8 (5.72)			–0.09	
		Hyperarousal	8.6 (5.92)			7.6 (5.30)			0.18			6.3 (4.89)				8.0 (5.29)			–0.33	
	PHQ-9^f^			10.7 (6.05)			8.0 (0.18)			0.47			8.6 (4.72)				9.4 (4.44)			–0.17
	GAD-7^g^			7.7 (3.58)			5.7 (4.21)			0.51			5.5 (3.18)				7.2 (4.45)			–0.44
	**EQ-5D-5L (index value)**			0.7 (0.19)			0.8 (0.18)			0.54			0.8 (0.13)				0.7 (0.08)			–0.93
		Mobility	1.7 (1.07)			1.3 (0.63)			0.45			1.3 (0.45)				1.1 (0.29)			0.53	
		Self-care	1.4 (0.67)			1.3 (0.65)			0.15			1.1 (0.29)				1.3 (0.45)			–0.53	
		Usual activities	2.3 (1.22)			1.9 (1.08)			0.35			1.8 (0.87)				2.0 (1.04)			–0.21	
		Pain/discomfort	2.2 (0.94)			1.8 (1.22)			0.36			1.4 (0.67)				2.3 (1.06)			–0.73	
		Anxiety/depression	2.7 (1.15)			2.3 (1.22)			0.34			1.8 (0.72)				2.3 (0.65)			–0.73	
	CPZ^h^ equivalent (mg/day)			488.5 (340.91)			462.3 (336.90)			0.08			413.9 (252.29)				386.8 (229.34)			0.11

^a^vCBTp: videoconference-based cognitive behavioral therapy for psychosis.

^b^UC: usual care.

^c^PANSS: Positive and Negative Syndrome Scale.

^d^BCI-J: Japanese version of the Beck Cognitive Insight Scale

^e^IES-R: Impact of Event Scale-Revised.

^f^PHQ-9: Patient Health Questionnaire-9.

^g^GAD-7: Generalized Anxiety Disorder-7.

^h^CPZ: chlorpromazine.

**Table 4:** Amount of change from baseline to posttest (N=24).

Variable				vCBTp^a^ + UC^b^ group (n=12), mean (SD)		UC group (n=12), mean (SD)	*t* test		*P* value
**Primary outcome**									
	PANSS^c^ (total score)			–9.5 (4.08)		6.9 (8.47)	6.05		<.001
**Secondary outcomes**									
	**PANSS (positive)**			–3.3 (2.99)		0.9 (1.98)	4.10		<.001
		Delusion	–0.8 (0.75)		0.1 (1.00)		2.31	.03	
		Conceptual disorganization	–0.3 (0.49)		0.3 (0.62)		0.56	.02	
		Hallucinatory behavior	–0.3 (0.75)		0.4 (0.79)		2.11	.046	
		Excitement	–0.5 (1.17)		0.0 (0.00)		1.48	.15	
		Grandiosity	–0.5 (1.17)		–0.1 (0.51)		1.13	.27	
		Suspiciousness	–0.9 (1.24)		0.2 (1.11)		2.25	.04	
		Hostility	–0.1 (0.29)		0.1 (0.29)		1.41	.17	
	PANSS (negative)			–1.2 (1.40)		2.7 (3.89)	3.21		.004
	**PANSS (general psychopathology)**			–5.0 (2.80)		3.3 (4.79)	5.20		<.001
		Anxiety	–0.8 (0.94)		0.0 (1.41)		1.70	.10	
		Guilt feeling	–0.8 (1.03)		0.2 (0.83)		2.61	.02	
		Depression	–0.5 (0.67)		0.8 (1.47)		2.86	.009	
		Lack of judgment and insight	–0.5 (0.52)		0.2 (0.58)		2.97	.007	
	BCIS-J^d^ (self-certainty)			–0.5 (1.83)		0.9 (3.37)	1.28		.21
	BCIS-J (self-reflectiveness)			2.2 (4.63)		1.3 (3.33)	–0.56		.58
	**IES-R^e^ (total score)**			–5.3 (14.87)		1.8 (16.65)	1.10		.28
		Intrusion	–3.3 (5.55)		–0.5 (6.16)		1.15	.26	
		Avoidance	–1.1 (7.03)		0.6 (9.00)		0.51	.62	
		Hyperarousal	–1.0 (4.57)		1.7 (5.00)		1.36	.19	
	PHQ-9^f^			–2.7 (5.33)		0.8 (4.22)	1.78		.09
	GAD-7^g^			–2.0 (4.71)		1.7 (3.52)	2.16		.04
	**EQ-5D-5L (index value)**			0.1 (0.15)		–0.1 (0.13)	–3.14		.005
		Mobility	–0.3 (0.89)		–0.2 (0.39)		0.60	.56	
		Self-care	–0.1 (0.79)		0.2 (0.58)		0.88	.39	
		Usual activities	–0.3 (0.89)		0.3 (0.87)		1.63	.12	
		Pain/discomfort	–0.4 (1.24)		0.8 (1.03)		2.69	.01	
		Anxiety/depression	–0.4 (0.79)		0.5 (0.67)		3.05	.006	
	CPZ^h^ equivalent (mg/day)			6.8 (22.61)		–27.1 (51.63)	–2.01		.006

^a^vCBTp: videoconference-based cognitive behavioral therapy for psychosis.

^b^UC: usual care.

^c^PANSS: Positive and Negative Syndrome Scale.

^d^BCI-J: Japanese version of the Beck Cognitive Insight Scale.

^e^IES-R: Impact of Event Scale-Revised.

^f^PHQ-9: Patient Health Questionnaire-9.

^g^GAD-7: Generalized Anxiety Disorder-7.

^h^CPZ: chlorpromazine.

### Secondary Outcomes

The mean change at posttest for the 3 PANSS subscales was as follows. The mean change in positive symptoms from baseline was –3.33 (95% CI –5.24 to –1.43) in the vCBTp plus UC group and 0.92 (95% CI –0.34 to 2.17) in the UC group. The difference between groups was significant at 4.25 (95% CI 2.10-6.40; *P*<.001). The mean change in negative symptoms was 2.67 (95% CI 0.19-5.14) in the UC group and –1.17 (95% CI –2.06 to –0.28) in the vCBTp plus UC group. The difference between groups was 3.83 (95% CI 1.36-6.31; *P*=.004). The mean change in overall psychopathology was 3.33 (95% CI 0.29-6.38) in the UC group and –5.00 (95% CI –6.78 to –3.22) in the vCBTp plus UC group. The difference between groups was 8.33 (95% CI 5.01-11.66; *P*<.001), with all 3 subscales showing significant differences.

The means and SDs of the secondary endpoints other than the PANSS and the mean changes are presented in Tables 3 and 4, respectively. There were no statistically significant differences between the 2 groups, including the subitems for BCIS-J, IES-R, PHQ-9, and CPZ. However, the vCBTp plus UC group had improved mean values for all change measures.

### Other Outcomes

When analyzing participants who showed a 20% or greater improvement in the PANSS total score as treatment responders, we observed that 4 participants (33%) in the vCBTp plus UC group responded positively to the treatment. In comparison, none (0%) in the UC group exhibited a 20% or greater improvement. When comparing the effect size of the mean change between the 2 groups in Table 3, the PANSS total score for the vCBTp plus UC group had Cohen *d*=1.45 (95% CI 3.96-15.04). Furthermore, the subscale effect sizes for the vCBTp plus UC group were as follows: PANSS positive symptoms, Cohen *d*=1.44 (95% CI 1.38-5.42); PANSS negative symptoms, Cohen *d*=0.53 (95% CI –0.72 to 3.12); and PANSS overall psychopathology, Cohen *d*=1.38 (95% CI 1.91-8.09). Notably, a significant effect was observed outside of negative symptoms.

At 0 weeks, the scores on the PHQ-9 [36] screening for suicidal ideation (item 9) were 3 points in 0 participants, 2 in 2 participants, 1 in 5 participants, and 0 in 17 participants. At 8 weeks, the scores were 3 points in 0 participants, 2 in 1 participant, 1 in 7 participants, and 0 in 16 participants. The patients were not excluded from the study because they were not at risk for imminent suicide as assessed by the researcher’s interviews and psychiatrist’s ratings.

During the session on trauma in the broadest sense, several participants began to cry as they recalled their painful past. However, none declined to participate in the study because of this.

As for safety evaluation, no serious adverse events occurred among the participants, including hospitalization. There were 2 adverse events in the UC group and 4 in the vCBTp plus UC group, none of which were attributable to vCBTp.

The total PANSS scores in the UC group worsened. The self-administered questionnaire, a secondary assessment, also showed worsening scores. The 8-week assessment of the UC group revealed changes in the participants’ life circumstances, including plans to retire due to poor physical and mental health, impending separation from a partner, difficulties in making friends at school, progressive illness in the family, and impending return to school after a leave of absence. The participants talked about the environmental changes in their lives.

## Discussion

### Principal Findings

This is the first pilot RCT to examine the efficacy of providing real-time and one-on-one vCBTp for patients with schizophrenia over 7 sessions. The study revealed that vCBTp was effective as an adjunct to UC with regard to the PANSS total score and each subscale at 8 weeks after the intervention.

### Comparison With Prior Work

In a study by Morrison et al [38], the PANSS total score improved in the group provided with pharmacotherapy and CBTp (compared with baseline) at 6 weeks, with an effect size of 0.66 (mean change of 6.06 points), and at 12 weeks, with an effect size of 1.04 (mean change of 12.36 points). In addition, although treatment guidelines recommend 16 or more sessions, a meta-analysis by Hazell et al [11] revealed that CBTp performed with 6 to 15 sessions still yielded a notable effect size of 0.46, indicating that a smaller number of sessions than the recommended 16 can be effective. Furthermore, a previous study of an intervention for self-esteem reported an effect size as large as 1.2 (n=18) for 7 sessions in an unformulated configuration [53]. This suggests that even fewer sessions may produce effects by clarifying target symptoms.

However, our study requires careful discussion because the significant difference in the PANSS score observed in our study involved not only an improvement in the vCBTp plus UC group but also a worsening in the UC group. Therefore, the effect size was not a comparison of the change in the 2 groups, but rather the change between week 0 and week 8 in the vCBTp plus UC group. The vCBTp plus UC group showed a significant effect size of 1.45 (mean change 9.5) in the total PANSS score at 8 weeks, exceeding that observed in previous studies. This exceeded the results observed in a previous study.

However, the scores themselves are not remarkable improvements, since the study by Morrison et al [38] showed a 12.36-point improvement in the PANSS total score at 12 weeks, whereas this study showed a 9.5-point improvement. The smaller SD of the vCBTp plus UC group in this study may have been one of the reasons for the large effect size.

It has been reported that the addition of CBT to pharmacotherapy resulted in an improvement of more than 20% in overall symptoms in 44.5% of participants [54]. In this study, 33% of participants in the vCBTp plus UC group had an improvement of 20% or more, resulting in a generally similar rate of improvement, although not as high as in previous studies.

The reasons for the improvement are discussed below. In the study by Naeem et al [39], which we referred to in setting our allocation adjustment factors, the PANSS score was 51.6 at baseline in the control group and 49.05 in the intervention group, which indicates mild symptoms. Again, the mean baseline scores for the vCBTp plus UC and UC groups were 54.2 and 52.3, respectively, indicating that participants had relatively mild to moderate symptom severity. Patients suitable for CBTp had mild to moderate psychiatric symptoms, and had a high level of insight [55], motivation to participate, cognitive insight [55], willingness to participate, and cognitive flexibility [56]. The following factors may have contributed to the improvement in the vCBTp plus UC group in this study. Participants with the ability to consent appropriately were included through the MacCAT-CR. This suggested that participants retained a certain level of comprehension and cognitive ability. Cognitive functioning is said to facilitate adaptation to CBTp [57]. It has also been reported that insights into the disease and mild symptoms predict good outcomes even after short-term CBTp sessions [58]. The participants in this study had mild symptoms and clear research consent, which may have contributed to the success of vCBTp.

Some participants had self-odor delusions, some were restricted from using transportation due to stress diarrhea symptoms, and some were not able to go outside due to a sense of forethought or paranoia. In addition, some patients struggled with the burden of transportation costs for travel. The ability to receive vCBTp at home seemed to reduce the labor burden associated with transportation.

After each session, a PDF summary of the session was sent to each participant, and the sessions were individualized to the case. The subjective assistance needs of patients with schizophrenia include the desire to be listened to by a professional [59]. There was a lot of positive feedback about the experience of being listened to in a one-on-one setting. Sessions included positive rapport building through listening and individual case formulation through collaborative demonstration work to develop strategies for coping with stress. The participants seemed to find it refreshing to discuss coping strategies with a specialist. After forming rapport in the first half of the session, the participants shared their painful experiences with the therapist in the second half of the session. These experiences included intrusive memories about stressful events related to the onset of psychosis, while taking into consideration their own distress. The participants then shared their experiences of working on themselves, which they said helped them to accept themselves as they are now. There were also reports of the effects of working on oneself [60]. For most of the participants, this was the first experience of talking to a therapist about a painful event in their past.

Traumatic stress symptoms have been reported in approximately 12.4% of patients with schizophrenia [61], suggesting an integrated model of trauma and schizophrenia [62]. Intervention attempts have also been reported [63]. In this study, the 5th and 6th sessions dealt with intrusive memories of stressful events related to the onset of psychosis, and were designed to help participants understand core beliefs, work on themselves, and reflect on coping strategies that have helped them overcome their painful past. Although there were no significant differences between groups on the IES-R, the mean change scores for the vCBTp + UC group, including the subscales, showed a trend toward improvement.

Significant improvements were observed in the PANSS subscale scores for positive symptoms (*P*<.001), negative symptoms (*P*=.009), and general psychopathology (*P*<.001). Improvements were also observed in the self-administered GAD-7 and EQ-5D-5L and the EQ-5D-5L subcomponents of pain/discomfort and anxiety/depression. In recent years, nearly half of patients with schizophrenia have comorbid anxiety disorders [64]. In this study, it is possible that vCBTp was effective for those with subjective anxiety and discomfort.

We next discuss exacerbations in the UC group. Schizophrenia has been noted for its disease characteristics, including symptom instability and agitation due to stress in daily life [65,66], and several patients in the UC group reported anger, hopelessness, depression, and anxiety at assessment due to daily work, friends and family, and other life circumstances during the study period. There have been reports of a lack of coping skills [67] and high sensitivity to stress and symptoms [68] with regard to daily life stress in schizophrenia. In the UC group, stress related to coping with interpersonal relationships and worries in daily life may have also affected symptoms and contributed to worsening outcomes [69]. Schizophrenia carries a high risk of relapse, with 30% of patients being readmitted within 6 months to 1 year [70]. This may indicate that patients with schizophrenia have difficulty with coping skills and stress management [71]. In this study, most of the secondary endpoints of self-administered questionnaires also showed worsening values. The worsening of anxiety/pain on the GAD-7 and EQ-5D-5L was particularly noticeable, suggesting that there are anxiety-based effects in daily life events.

We initially estimated a dropout rate of 10%. This was considered an appropriate estimate considering that a previous study reported an average dropout rate of 13% [72] and another study reported a dropout rate of 11% [40]. However, there were no dropouts in this study. There are several possible reasons for this. First, this study consisted of fewer sessions than the formulated number of CBTp sessions. Some meta-analyses have shown that the dropout rate from research trials among patients with schizophrenia increases as the number of intervention sessions increases [73]. In addition, the implementation of MacCAT-CR with the inclusion criteria and the inclusion of participants who had firm research consent and understanding may have been some of the reasons for the lack of dropouts. Furthermore, many participants were motivated to participate in the study because they commented that they wanted to see more treatment options for schizophrenia and they wanted to help others who were suffering like they were. It has been reported that careful explanation of the methods and transparency of the study can increase motivation to participate and decrease the dropout rate [74]. As the participants had never received CBT before, there may have been some novelty to the study. In any case, it was inferred that they were highly motivated to participate. These factors could be the reasons for the absence of dropouts.

PHQ-9 item 9 was screened and used as a safety measure. Attention to suicidal ideation is important when the subject has schizophrenia, as a study [75] validating the risk of suicidal behavior in psychosis with responses to item 9 of the PHQ-9 stated that the risk is higher for psychosis than depression. In this study, the number of participants who scored more than 1 point was also higher. Some participants in this study also scored 1 or more, but when interviewed afterward, they said that they scored based on their mood of anxiety and discouragement, saying that they feel sad or painful enough to want to die, and no one showed real suicidal thoughts. None of the adverse events were attributable to the vCBTp intervention (eg, cold and reflux esophagitis), and there were no serious adverse events, confirming a certain level of safety.

The use of videoconference systems can reduce the burden on the patient’s hospital visit effort, that is, the financial burden of transportation costs, weather effects, and social fears associated with transportation use. It can also reduce the burden on CBTp providers. As a result, accessibility to CBTp could be improved.

Regarding remote interventions, it has been reported that simply providing devices is not sufficient to address digital exclusion, and training in using the devices is likely to be required [76]. However, with a simple structure, pretraining, and a support system, such as in this study, which can allow patients to open an email and tap a URL to participate in a videoconference, it may be possible to implement psychosocial interventions remotely using digital technology for patients with schizophrenia.

The major originality of this study is that participants could receive CBTp in the comfort of their own homes. Currently, self-help approaches using digital media, such as the internet and cell phones, are being actively developed [77,78]. As a result, many patients are interested in using digital technology to improve their mental health [25]. Low-intensity interventions may benefit many patients with schizophrenia without increasing costs [79]. Furthermore, studies have demonstrated the effectiveness of targeted CBTp with reduced session frequency [11,12,14]. Furthermore, an online intervention via videoconferencing has been reported to be feasible for patients with schizophrenia [80]. These considerations suggest that the provision of vCBTp using a remote format with reduced session frequency may be feasible enough for implementation in practice, especially in terms of a stepped care model to deliver CBTp [8,81].

### Limitations

This exploratory study had some limitations. First, the UC settings varied, and these were not subject to comparison or analysis between the 2 groups. The frequency of outpatient visits varied from once a week to once every 2 months. The frequency and duration of use of psychiatric rehabilitation facilities also varied. These may have influenced outcomes as confounding variables. Prescription medications and methods of administration also varied. The study did not analyze the influence of external variables such as the participants’ living environments; thus, future research on correlations with external variables would be desirable.

Second, it was not clear what targets the vCBTp program influenced. Although various points were considered, the primary mechanism of change was unclear because the study incorporated a variety of techniques.

Third, due to the absence of a follow-up assessment, the determination of efficacy remained unclear. Therefore, a 1-year follow-up is being conducted for vCBTp participants as an additional study.

Fourth, the EQ-5D-5L tool was used as a subjective measure of quality of life but was not validated for quality-adjusted useful life or cost-effectiveness. Future studies should examine the contribution of the vCBTp program to health care costs.

Fifth, the sample size in our pilot study was relatively small. Since improvements were observed in this study, a larger multi-center study is desired.

Finally, since this trial was conducted with only 1 therapist and 1 assessor, we are yet to examine how to share the experience of this vCBTp intervention with staff at other facilities. It is important to consider the possibility of social implementation in the future.

### Conclusions

The findings of this RCT indicate that vCBTp is effective in treating symptoms over 7 sessions. The findings show that vCBTp can be effective at a distance and with a reduced number of sessions, which can reduce the burden on the practitioner and improve the accessibility of CBTp for patients. It may also contribute to relapse prevention and psychosocial support during stepped care. Further investigations are required to determine the durability of this effect.

## Appendix

Multimedia Appendix 1 CONSORT eHEALTH checklist (V 1.6.1).

## Abbreviations

BCIS-J: Japanese version of the Beck Cognitive Insight Scale
CBTp: cognitive behavioral therapy for psychosis
CONSORT: Consolidated Standards of Reporting Trials
CPZ: chlorpromazine
GAD-7: Generalized Anxiety Disorder-7
IES-R: Impact of Event Scale-Revised
MacCAT-CR: MacArthur Competence Assessment Tool for Clinical Research
NICE: National Institute of Health and Clinical Excellence
PANSS: Positive and Negative Syndrome Scale
PHQ-9: Patient Health Questionnaire-9
RCT: randomized controlled trial
UC: usual care
vCBTp: videoconference-based cognitive behavioral therapy for psychosis

## Data Availability

The data are available upon reasonable request.
